# What Will We Learn if We Start Listening to Women with Menses-Related Chest Pain?

**DOI:** 10.3390/jcm14092882

**Published:** 2025-04-22

**Authors:** Tomasz Marjanski, Aleksandra Czapla, Julia Niedzielska, Lena Grono, Jagoda Bobula, Renata Świątkowska-Stodulska, Ewa Milnerowicz-Nabzdyk

**Affiliations:** 1 Department of Thoracic Surgery, Faculty of Medicine, Medical University of Gdansk, 80-214 Gdansk, Poland; marjanski@gumed.edu.pl; 2 Department of Endocrinology and Internal Diseases, Faculty of Medicine, Medical University of Gdansk, 80-214 Gdansk, Poland; aleksandraczapla@gumed.edu.pl (A.C.); renata.swiatkowska-stodulska@gumed.edu.pl (R.Ś.-S.); 3 Department of Gynecology, Obstetrics and Neonatology, Medical University of Gdansk, 80-211 Gdansk, Poland; lenagrono@gumed.edu.pl (L.G.); jagoda.bobula@gumed.edu.pl (J.B.); 4 Clinical Department of Gynecological Oncology, Centre of Oncology, Medical Faculty, Opole University, 45-758 Opole, Poland; ewa.milnerowicz@wp.pl

**Keywords:** endometriosis, chest pain, catamenial pneumothorax, symptoms, catamenial pain, catamenial hemoptysis, women

## Abstract

**Background**. Thoracic endometriosis is thought to be the most common form of endometriosis occurring outside of the pelvis. We aimed to characterize thoracic symptoms of endometriosis in a population of patients not necessarily suffering from catamenial pneumothorax, which is most commonly identified as a symptom of thoracic endometriosis. **Material and methods**. We used a web-based survey addressed to users of two Polish endometriosis patient advocate organizations. The factor that qualified patients for the study was the presence of symptoms in the chest related to the menstrual cycle. **Results**. A total of 92 respondents were questioned. In this group, 96% (88/92) of patients were previously diagnosed with pelvic endometriosis, 20% (18/92) with thoracic endometriosis, and 18% (17/92) with diaphragmatic endometriosis. The percentage of patients diagnosed with both thoracic and diaphragmatic endometriosis was 15% (14/92). Ninety-eight percent of patients suffered from pain. The four most common symptoms reported by patients were chest pain, dyspnea, cough, and stunned limb, occurring in 96%, 67%, 52%, and 33%, respectively. The feeling of a stunned, weakened limb occurs in older women at 38.4 vs. 35.5 years of age (*p* = 0.021). There is a trend that women who suffer pain (36.7 vs. 31.3 years of age *p* = 0.053) and hemoptysis (41.0 vs. 36.2 years of age *p* = 0.059) are older than women without these symptoms. We identified two unique symptoms of thoracic endometriosis—pouring liquid sensation (13%) and popping sensation (12%)—which can be related to a small amount of gas and fluid in the pleural cavity. **Conclusions**. Patients who have endometriosis suffer from a constellation of thoracic symptoms related to menses.

## 1. Introduction

Endometriosis is defined as the presence of endometrial-like glands and/or stroma out of the uterus [1]. As the body of evidence grows, there is a diminishing area of controversy concerning the diagnosis and treatment of pelvic and abdominal endometriosis [2,3,4,5]. Nevertheless, there are still some existing difficulties in defining, diagnosing, and treating extrapelvic endometriosis [6,7,8,9,10,11]. Endometriosis can be found in extrapelvic locations in 9–15% of patients, and thoracic endometriosis is thought to be the most common form of endometriosis occurring outside of the pelvis, being present in 0.7–4.7% of patients [11,12]. Indubitably, the most typical symptom of thoracic endometriosis is catamenial pneumothorax [10,13,14]. The high number of patients suffering from catamenial pneumothorax suggests that the actual prevalence of symptomatic pneumothorax may be underestimated and highlights the need to re-evaluate its proportion in comparison to other clinical manifestations of endometriosis. Diaphragmatic endometriosis and thoracic endometriosis are frequently treated as separate entities in the literature, yet they are interconnected. Additionally, it should be noted that the cause of catamenial pneumothorax is complex and typically involves diaphragmatic endometriosis. The typical symptoms of both diaphragmatic and thoracic endometriosis should be considered in every patient with pelvic endometriosis [11,15]. Despite the usually relatively large mass of endometrial implants in the pelvic or peritoneal area, the majority of extrapelvic seedings are minimal and often represented by deficient pathological patterns [16]. Thoracic endometriosis presents a broad range of ambiguous clinical symptoms, commonly known as thoracic endometriosis syndrome (TES). The primary manifestations of TES are believed to include catamenial pneumothorax, hemothorax, hemoptysis, and pulmonary nodules [7,12,17,18,19]. We suggest that other, less specific symptoms, such as catamenial chest or shoulder pain that may radiate to the neck, periscapular region, and abdomen [7,17], might be more prevalent. In rare cases, hydrothorax is reported as the predominant symptom in women with thoracic endometriosis [20].

The clinical presentation of diaphragmatic endometriosis is commonly mentioned in the sections of scientific papers describing thoracic endometriosis, which is clearly justified by the fact that the symptoms of diaphragmatic endometriosis occur nearly exclusively in the upper part of the body. We aimed to characterize thoracic symptoms of endometriosis in a population of patients not necessarily suffering from catamenial pneumothorax.

## 2. Materials and Methods

We used a web-based survey addressed to users of two Polish patient advocate organizations whose main focus is endometriosis: Fundacja Pokonać Endometriozę (Eng. Foundation Conquer Endometriosis) and Odmosfera (Eng. The Sphere of Pneumothorax). The participants were reached through Meta Platforms, Inc. (Menlo Park, CA, USA; formerly Facebook, Inc.). The Polish questionnaire was administered using Google Forms (Google LLC, Mountain View, CA, USA), an online survey tool. Since Google Forms does not provide a version number, the homepage of the software is available at https://docs.google.com/forms (accessed on 10 February 2024). The original questionnaire in Polish and its English transcription are available in Supplementary Materials File S1. The main areas of the questionnaire were as follows:

1. General information—age, medical history of endometriosis, including family history, and presence of chest pain during menstrual cycle. Patients were also asked whether they had been previously diagnosed with pelvic and/or thoracic and/or diaphragmatic endometriosis, and how the diagnosis had been obtained (‘intraoperatively’, ‘radiologically’, ‘other test’, or ‘not applicable’).
2. Fertility-related section—history of infertility and pregnancies, including miscarriages.
3. Status of hormonal therapy—history of hormonal therapy treatment, hysterectomy and adnexectomy.
4. Symptoms present during the menstrual cycle—including chest pain, dyspnea, cough, hemoptysis, numbness of a limb, and sensation of irregular heartbeat, during the last 6 menstrual cycles. In the case of receiving hormonal therapy, patients were asked to refer to the period prior to treatment. Patients were also able to provide information about other presented symptoms and the exact age of their onset.
5. Regularity of symptoms during menstruation—patients were asked to assess the regularity of previously mentioned symptoms (from 1 to 6 times, or not applicable) during the last 6 menstruations.
6. Regularity of symptoms during ovulation—patients were asked to assess the regularity of previously mentioned symptoms (from 1 to 6 times, or not applicable) during the last 6 ovulations. Information that ovulation usually occurs on the 14th day of the cycle was provided.
7. Additional questions about chest pain during the menstrual cycle—history of cholelithiasis, correlation with meals or dietary mistakes.
8. Characteristics of chest pain during menstrual cycle—pain intensity, pain frequency, painkiller usage, pain-related physical activity limitation, aggravating and alleviating factors.
9. Pain location—patients were asked to graphically depict the areas that become painful during the menstrual cycle, using a provided dermatome map. Precise instructions were included concerning methods of marking the painful areas and submitting the scheme.
10. Comment section—patients could leave additional remarks and comments regarding their condition.

In order to both ensure clarity for the patients and improve the quality of our research, a precise yet easily comprehensible definition of endometriosis and thoracic endometriosis was presented at the beginning of the questionnaire. Endometriosis was defined as ‘the presence of ectopic endometrial tissue (glands and stroma) outside the uterine cavity’. Thoracic endometriosis was defined as ‘involving changes in the lungs, pleural cavity, diaphragm, and bronchi, confirmed through histopathological examination of specimens (obtained via aspiration, thoracotomy, or bronchoscopy)’.

The analysis was performed using STATA 18.0. Unpaired data, characterized by a normal distribution, was compared with an unpaired *t*-test. In the case of non-normal distribution, a Mann–Whitney U-test was applied to compare two unmatched samples. The χ^2^ test was used for comparison of categorical variables. The accepted level of *p*-value was 0.05. Relative risks (RRs) were calculated with a 95% confidence interval (CI). For RR calculation, the data concerning the occurrence of symptoms were recoded to 0 if the symptoms were not occurring or occurring 1–2 times during the last 6 months and recoded to 1 if the symptoms occurred between 3 and 6 times during the last 6 months. The models of linear regression were built to assess the influence of factors (side of the disease, duration of symptoms, age of the diagnosis of endometriosis) on the occurrence of pain, dyspnea, hemoptysis, cough, the feeling of a stunned limb, and heart arrhythmia during last 6 months, coded as a continuous value. Meaningful factors were identified after stepwise backward elimination of factors with *p*-value > 0.2. Logistic regression models were built to evaluate the effect of the duration of symptoms on the possibility of reducing the symptoms by different factors.

## 3. Results

A total of 92 respondents were questioned. In Table 1, clinical characteristics of patients are presented. In our cohort, 96% (88/92) of patients had been previously diagnosed with pelvic endometriosis, 20% (18/92) with thoracic endometriosis, and 18% (17/92) with diaphragmatic endometriosis. The percentage of patients diagnosed with both thoracic and diaphragmatic endometriosis was 15% (14/92). The factor that qualified patients for the study was the presence of symptoms in the chest related to the menstrual cycle. In fact, 98% of patients suffered from pain. As the questionnaires were distributed among members of support groups for endometriosis patients, this obviously contributed to the fact that 96% of patients had a previous diagnosis of endometriosis. A relatively large proportion of respondents were diagnosed with thoracic and diaphragmatic endometriosis, which is another consequence of recruiting the respondents from the thoracic endometriosis patient support group. Most of the patients were diagnosed on the basis of pathological examination of specimens harvested during diagnostic laparoscopy. About one-third (32%) were diagnosed on the basis of radiological examination. Part of the table briefly characterizes problems with fertility among respondents. Lastly, 5% of patients were diagnosed with gallstones, and in total, 9% of patients related the presence of symptoms to food intake.

In Table 2, mean ages of the groups of women with and without symptoms of thoracic endometriosis syndrome are presented. The four most common symptoms reported by patients were chest pain, dyspnea, cough, and stunned limb, occurring in 96%, 67%, 52%, and 33%, respectively. Other symptoms reported by the patients occurred in not more than 13% of them. The feeling of a stunned, weakened limb occurs in older women at 38.4 vs. 35.5 years of age (*p* = 0.021). There is a trend that women who suffer pain (36.7 vs. 31.3 years of age *p* = 0.053) and hemoptysis (41.0 vs. 36.2 years of age *p* = 0.059) are older than women without these symptoms. We identified two unique symptoms of thoracic endometriosis—pouring liquid sensation (13%) and popping sensation (12%)—which can be related to a small amount of gas and fluid in the pleural cavity.

We tested the relative risk of symptoms associated with pain during menstruation. The patients who have chest pain during menstruation have a 28% higher risk of coexisting cough (RR: 1.289; 95%CI: 1.080–1.539; *p* = 0.007) than those without pain, 30% higher risk of coexisting dyspnea (RR: 1.303; 95%CI: 1.070–1.586; *p* = 0.005), 29% higher risk of coexisting stunned limb (RR: 1.2963; 95%CI: 1.141–1.147; *p* = 0.013), and 39% higher risk of coexisting heart arrhythmia (RR: 1.396; 95%CI: 1.114–1.748; *p* < 0.001). The patients who experience thoracic pain during menstruation do not have a significantly higher risk of hemoptysis (RR: 1.216; 95%CI: 1.104–1.339; *p* = 0.511).

All patients with confirmed thoracic endometriosis presented chest pain during menstruation (RR: n/a; 95%CI: n/a; *p* = 0.030). Patients with confirmed thoracic endometriosis, compared to those without a diagnosis of thoracic endometriosis, similarly often experienced some symptoms during menstruation: cough (RR: 1.698; 95%CI: 0.739–3.906; *p* = 0.207), dyspnea (RR: 1.250; 95%CI: 0.542–2.881; *p* = 0.599), hemoptysis (RR: 2.647; 95%CI: 0.620–11.290; *p* = 0.272), and stunned limb (RR: 1.590; 95%CI: 0.676–3.740; *p* = 0.296).

In logistic regression, the duration of symptoms did not have an impact on alleviating the symptoms with medications (OR: 0.968; 95%CI: 0.896–1.048; *p* = 0.432), exercise (OR: 1.066; 95%CI: 0.971–1.170; *p* = 0.179), rest (OR: 1.017; 95%CI: 0.940–1.101; *p* = 0.670), warm compress (OR: 1.048; 95%CI: 0.960–1.144; *p* = 0.293), body position (OR: 0.958; 95%CI: 0.881–1.040; *p* = 0.304), or other measures (OR: 1.032; 95%CI: 0.91401.167; *p* = 0.606). Similarly, the duration of symptoms did not have any impact on the intensity of symptoms (medications: OR: 1.000; 95%CI: 0.935–1.069; *p* = 1.000; exercise: OR: 1.087; 95%CI: 0.992–1.191; *p* = 0.073; stress: OR: 0.990; 95%CI: 0.887–1.107; *p* = 0.871; deep breaths: OR: 1.034; 95%CI: 0.926–1.155; *p* = 0.551; body position: OR: 1.039; 95%CI: 0.966–1.117; *p* = 0.300; other: OR: 0.937; 95%CI: 0.842–1.043; *p* = 0.233; nothing: OR: 0.968; 95%CI: 0.828–1.133; *p* = 0.689).

## 4. Discussion

**Figure 1:**
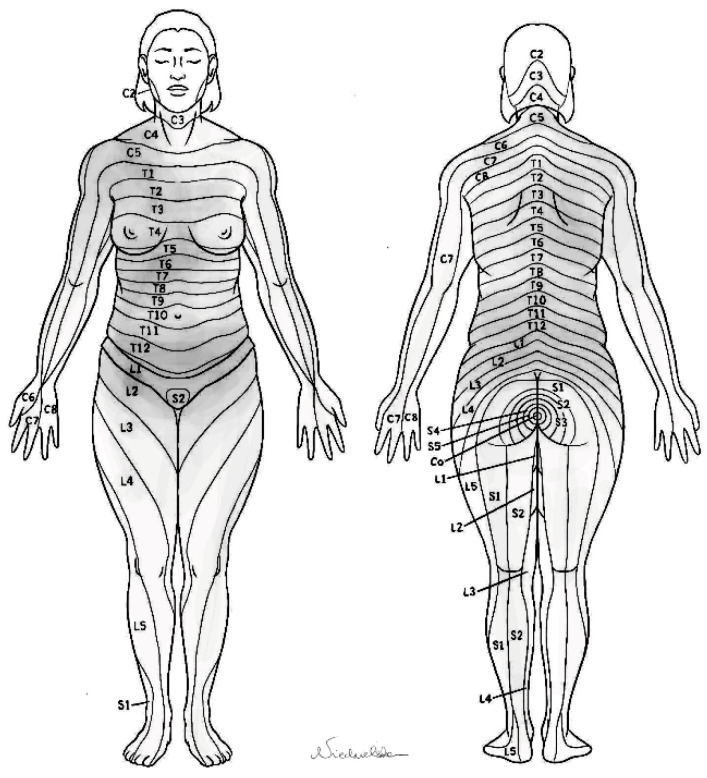
Overlaid patterns of frequency of pain in 32 participants of the survey who completed this part.

In our study, the presence of pain (98%) was much more common than other symptoms like hemoptysis or dyspnea, which was also reported by other authors [10]. We conclude that the occurrence of chest pain in patients with endometriosis is underestimated. In this paper, we made an attempt to characterize this condition more accurately. We identified original symptoms, which we called popping sensation and pouring liquid sensation. These are not common (12–13%, respectively) in the study population. Additionally, these symptoms are not frequently reported to respiratory specialists in diseases other than thoracic endometriosis. We would like to raise the question of whether those symptoms have been recorded by clinicians elsewhere. The presence of these symptoms is usually associated with recurring catamenial pneumothorax and may be a result of coexisting air and fluid in the pleural cavity. The distribution and frequency of pain is presented in Figure 1.

The right-sided predominance of the mentioned symptoms is associated with the clockwise peritoneal fluid movement that carries endometrial cells; it makes ectopic endometrial tissue more prone to being implanted in the right hemidiaphragm, rather than the left one, where the flow is additionally hindered by the falciform ligament [21,22,23]. Even though the symptoms are believed to be mostly catamenial (occurring between 24 h before and 72 h after the onset of menses), the correlation is not an axiom, as both coexist with other phases of the menstruation cycle and few asymptomatic cases have been reported [7,24]. In our study, we found that surprisingly only 42% of those surveyed suffered from exclusively right-sided symptoms, whereas 37% had bilateral and 21% had left-sided manifestations. It is difficult to comment on this due to the fact that pneumothorax related to thoracic endometriosis occurs in 85–95% of cases exclusively in the right pleural cavity [13]. One possible explanation is that the chest pain occurring during menses or less commonly during ovulation may be related not only to diaphragmatic endometriosis but also to the potential presence of deep-infiltrating pelvic endometriosis. The presence of significant foci of endometriosis infiltrating viable structures of the pelvis and abdominal wall may lead to radiating pain to the lower parts of the chest. From the authors’ experience (TM, EMN), some of the patients treated by extensive debulking endometriosis surgery in the pelvis may also experience some relief as far as their thoracic symptoms are considered. The considerable range of different painful areas (bilaterally in the chest and pelvis) visualized in Figure 1 may indicate a common and clinically meaningful coexistence of symptomatic thoracic and pelvic endometriosis.

The presence of the innervation of the diaphragm from the phrenic nerve and T5–T11 intercostal nerves may be the reason for the common coexistence of the areas of referred pain in the shoulder, arm, neck, and inframammary area. Diaphragmatic palsy is a unique form of thoracic endometriosis [25]. The estimated prevalence of endometriosis among women of reproductive age reaches up to 10% [26]. On the other hand, diaphragmatic endometriosis may be challenging to adequately confirm in radiological examinations with a sensitivity of 78–83%, but only in experienced centers [27]. Those require careful differentiation of the referred pain with rotator cuff tendinopathy, adhesive capsulitis, other arthropathies, cervical spine disorders, cholelithiasis, and neoplasms including Pancoast tumors, chest wall tumors, and others. In our group, the presence of gallstones was as low as 5%, and only 9% of those surveyed linked their symptoms with food intake. Diagnosing TES may be challenging due to overlapping symptoms of other diseases mentioned above [28].

The median age of the patients filling out the questionnaire was 37 years, while they had their diagnosis of endometriosis at a median age of 31.5. This reflects the typical pattern of thoracic endometriosis as a late consequence of endometriosis [13].

Any of the aforementioned symptoms present in women of reproductive age should raise suspicions and lead the patient on a path of thorough diagnostic examination. It should also be commonly discussed during routine investigations of women diagnosed and treated for pelvic endometriosis [15]. The following features have been recognized to be particularly common and can be treated as diagnostic criteria: (1) involvement of the right hemithorax; (2) temporal, cyclic coexistence of pneumothorax at the onset of menses; (3) lack of pneumothorax during other phases of the menstrual cycle; (4) occurrence of the disease approximately in the fourth decade of life; and (5) a lack of pneumothorax while pregnant or taking ovulation inhibitors [29]. On the other hand, the presence of pneumothorax during vaginal labor may be a consequence of thoracic endometriosis [30]. We believe that clinical presentation is the key criterion for the diagnosis of thoracic endometriosis. Nonetheless, derogations from these criteria do not exclude the possibility of thoracic endometriosis [22]. Insufficient sensitivity of radiological procedures and typical clinical presentation lead to the necessity of diagnosing diaphragmatic endometriosis without pathological confirmation. It is important to underline that the presence of chest pain or other thoracic symptoms, related to the menstrual cycle, occurring in a woman with suspected or confirmed pelvic endometriosis should be a weak criterion for diagnosing thoracic endometriosis. In highly symptomatic patients, minimally invasive surgery remains an important diagnostic technique of TES since it both visualizes the endometrial implants and allows their simultaneous resection [31,32]. Simultaneous thoracoscopy and laparoscopy while the patient is positioned in lateral decubitus positions allow for optimal visualization and treatment of diaphragmatic endometriosis, especially on the right side [7], and should be recommended. The presence of endometrial lesions on the diaphragm or lung, as well as the presence of diaphragmatic perforations, is postulated to be a strong criterion for the diagnosis of thoracic endometriosis. Other pathological diaphragmatic perforations (Bochdalek’s hernia, Morgagni’s hernia) present very differently from the “Swiss cheese holes” in the membranous part of the diaphragm.

The authors proposed that a prolonged duration of symptoms might lead to certain alterations, such as the alleviation or intensification of symptoms, which have not been previously documented in the medical literature. However, this hypothesis was not supported by the logistic regression analysis in our study. Consequently, we suggest that similar interventions may be appropriate for both early and persistent stages of the disease. Pharmacotherapy demonstrates comparable efficacy to other treatment methods. Additionally, our findings did not indicate that factors such as stress, exercise, or body position more frequently exacerbate symptoms in cases of long-standing thoracic endometriosis syndrome (TES).

The study has significant limitations. This is a voluntary survey of an unrestricted population with access to social media in Poland. It does not allow a prompt generalization of the results of the study due to selection biases. The patients describe their symptoms in the survey; however, no objective verification is provided. Obtaining medical data has two aspects of lack of verification. Firstly, there was no formal physical examination which may lead to information biases. Secondly, the medical diagnosis remained unconfirmed as the medical files were not collected, which is another source of plausible information biases. We would like to emphasize that the nature of this voluntary study may lead to response biases because the respondents may modify their responses to underline the significance of their symptoms. It cannot be definitively ruled out that overlapping symptoms of other diseases may have affected the interpretation of the results of this study.

The authors contend that this paper could significantly contribute to the development of diagnostic criteria for identifying thoracic endometriosis syndrome (TES) without the need for pathological confirmation, thereby facilitating earlier pharmacotherapy and reducing the necessity for unwarranted diagnostic surgeries. We strongly express a plea to consider diaphragmatic endometriosis as an important (if not the most important) form of thoracic endometriosis. If we identify thoracic endometriosis as a condition requiring multi-specialist treatment, then it is important to accept common definitions to enable appropriate education and feasible cooperation. Lack of such arrangements will lead to further deprivation of a large proportion of patients from adequate forms of available treatment. Well-designed database studies may overwhelm the limitations of this study and reveal the true incidence and clinical presentation of thoracic endometriosis.

## 5. Conclusions

The occurrence of chest pain is underestimated and much more common than hemoptysis or dyspnea in patients with endometriosis. Chest pain in patients with endometriosis does not have to be right-sided. Cough, hemoptysis, dyspnea, and feeling of a stunned limb are common in patients with chest-related symptoms of endometriosis.

## Figures and Tables

**Table 1:** General characteristics of study population.

Clinical Presentation	Clinical Presentation
Age	Median 37 years (22–48)
Diagnosed with endometriosis	96% (88/92)
Diagnosed with thoracic endometriosis	20% (18/92)
Diagnosed with diaphragmatic endometriosis	18% (17/92)
Method of diagnosis of endometriosis: Intraoperative Radiological Other and not applicable	53% (49/92) 32% (23/92) 21% (19/92)
Age of diagnosis of endometriosis	31.5 (18–46)
Presence of chest pain during menstrual cycle	98% (90/92)
Presence of other symptoms but not chest pain or hemoptysis during menstrual cycle	2% (2/92)
Laterality of symptoms Right Left Bilateral	42% (39/92) 21% (19/92) 37% (34/92)
Family history of endometriosis	25% (23/92)
Fertility I did not have problems with getting pregnant I had problems with getting pregnant Not applicable	59% (54/92) 21% (19/92) 21% (19/92)
Treated for infertility	34% (31/92)
How many times was pregnant 0 1 2 3 or more	30% (28/92) 34% (31/92) 24% (22/92) 12% (11/92)
How many miscarriages 0 1 2 3 or more	76% (70/92) 16% (15/92) 5% (5/92) 2% (2/92)
Current hormonal treatment of endometriosis	57% (40/70)
Surgical treatment for pelvic endometriosis Adnexectomy Other procedures	13% (9/71) 87% (62/71)
Diagnosed with gallstones	5% (5/92)
Association of symptoms with food intake	8% (7/92)

**Table 2:** Age comparison in patients with or without symptoms during menstruation.

Symptoms During Menstruation	Number of Patients with Symptom (%)	Mean Age in Women with Pain [Years] (SD)	Number of Patients Without Pain (%)	Mean Age Women Without Symptom [Years] (SD)	*p*-Value
Chest pain	88 (96%)	36.7 (5.2)	4 (4%)	31.3 (10.9)	0.053 *
Shoulder pain	8 (9%)	36.6 (3.2)	84 (91%)	36.5 (5.7)	0.933
Scapular pain	10 (11%)	38.7 (4.3)	82 (89%)	36.2 (5.6)	0.179
Arm pain	4 (4%)	35.6 (2.2)	88 (96%)	36.3 (5.6)	0.656
Neck pain	4 (4%)	36.5 (6.9)	88 (96%)	36.5 (5.5)	0.990
Numbing	2 (2%)	33.5 (0.7)	90 (98%)	36.5 (5.6)	0.447
Dyspnea	62 (67%)	36.7 (5.6)	30 (33%)	36.1 (5.4)	0.609
Cough	48 (52%)	36.6 (5.1)	44 (58%)	36.3 (6.0)	0.749
Hemoptysis	5 (5%)	41.0 (5.1)	87 (95%)	36.2 (5.5)	0.059 *
Tension in the chest	4 (4%)	36.5 (5.1)	88 (96%)	36.5 (5.6)	0.995
Stunned limb	30 (33%)	38.4 (5.4)	62 (67%)	35.5 (5.4)	0.021 **
Pouring liquid sensation	12 (13%)	38.8 (5.3)	80 (87%)	36.1 (5.5)	0.126
Popping sensation	11 (12%)	38.5 (4.6)	81 (88%)	36.2 (5.6)	0.186
Pressure	2 (2%)	37.0 (4.2)	90 (98%)	36.4 (5.6)	0.892
Pressure and weight	7 (8%)	37.6 (4.5)	85 (92%)	36.4 (5.6)	0.586

* marks the *p*-values below 0.05 level and ** marks the *p*-values close to the level of statistical significance.

## Data Availability

Source data are available on request.

## Supplementary Materials

The following supporting information can be downloaded at: https://www.mdpi.com/article/10.3390/jcm14092882/s1, File S1: Questionnaire_english_polish.
