# Supplementary material for: What Will We Learn if We Start Listening to Women with Menses-Related Chest Pain?
Source: J Clin Med. 2025 Apr 22;14(9):2882. doi: 10.3390/jcm14092882 (PMC12072188; doi:10.3390/jcm14092882)
Supplement: Supplementary file 1 [file jcm-14-02882-s001.zip › jcm-3515579-supplementary.pdf]

QUESTIONNAIRE – ENGLISH VERSION

We are a research team from the Medical University of Gdansk, Poland, conducting a study on chest pain in thoracic endometriosis. We created a questionnaire, and we aim to use it as a source of clinical data to write a publication describing this phenomenon.
Our primary goal is to improve the diagnostic process of thoracic endometriosis and increase awareness about this condition among both patients and clinicians.

Consequently, we kindly ask you to fill out the questionnaire. The study is entirely anonymous, and it should take a maximum of 10 minutes. We deeply believe that together we can contribute to improving the quality of life of many patients around the world.

Before following up with the questionnaire, please familiarize yourself with the definitions of endometriosis:
1. Endometriosis is defined as the presence of ectopic endometrial tissue (glands and stroma) outside the uterine cavity.
2. Thoracic endometriosis involves changes in the lungs, pleural cavity, diaphragm, and bronchi, confirmed through histopathological examination of specimens (obtained via aspiration, thoracotomy, or bronchoscopy).

For any technical issues or questions regarding the questionnaire, please feel free to contact us at the email address: endoquestmug@gmail.com

1. How old are You? Please provide only the number.

2. Do you experience any chest pain during your menstrual cycle?
   a) Yes
   b) No

3. On which side do You experience chest pain during your menstrual cycle?
   a) Right side
   b) Left side
   c) Both sides
   d) Not applicable

4. Have You been diagnosed with endometriosis?
   a) Yes
   b) No

5. Have You been diagnosed with thoracic endometriosis?
   a) Yes
   b) No

6. Have You been diagnosed with diaphragmatic endometriosis?
   a) Yes
   b) No

7. How were You diagnosed with endometriosis?
   a) Intraoperatively
   b) Radiologically
   c) Other test
   d) Not applicable

8. How old were You when the diagnosis was made? Please provide only the number. If you are not diagnosed with endometriosis, please leave this field blank.

9. Have there been confirmed cases of endometriosis in Your family?
   a) Yes
   b) No

FERTILITY-RELATED QUESTIONS.

10. Have You ever had difficulties getting pregnant?
    a) Yes
    b) No
    c) I have never tried

11. Have You ever been treated for infertility?
    a) Yes
    b) No

12. How many times were You pregnant?

13. How many miscarriages have You experienced?

HORMONAL THERAPY.

14. Do you receive hormonal therapy?
    a) Yes
    b) No

15. If yes – since when?

16. Have you undergone any of the following procedures?
    a) Hysterectomy (removal of uterus)
    b) Adnexectomy (removal of fallopian tubes and ovaries)
    c) None of the above

SYMPTOMS DURING THE MENSTRUAL CYCLE.

If you receive hormonal therapy and:

a) have no symptoms ⇨ please refer to the fully symptomatic period, before hormonal therapy

b) still have symptoms ⇨ please refer to the current symptoms.

17. Have you experienced any of the following symptoms during the last 6 menstrual cycles? Please check all that apply.
    a) Chest pain
    b) Dyspnoea (shortness of breath)
    c) Cough
    d) Haemoptysis (coughing up blood)
    e) Numbness of a limb
    f) Sensation of irregular heartbeat
    g) Not applicable

18. Other non-specific chest symptoms during the last 6 menstrual cycles (e.g. 'popping' sensation, fluid overload). If not applicable, please leave this field blank.

19. At what age did the mentioned symptoms start? Please provide only the number. If not applicable, please leave this field blank.

REGULARITY OF SYMPTOMS DURING MENSTRUATION.

During the last 6 menstrual cycles, how many times did you experience the following symptoms during menses (period)? The next section will address ovulation, so please focus only on menses in this part.

20. Chest pain:
    a) 1 time
    b) 2 times
    c) 3 times
    d) 4 times
    e) 5 times
    f) 6 times
    g) Not applicable

21. Dyspnoea (shortness of breath):
    a) 1 time
    b) 2 times
    c) 3 times
    d) 4 times
    e) 5 times
    f) 6 times
    g) Not applicable

22. Cough:
    a) 1 time
    b) 2 times
    c) 3 times
    d) 4 times
    e) 5 times
    f) 6 times
    g) Not applicable

23. Haemoptysis (coughing up blood):
    a) 1 time
    b) 2 times
    c) 3 times
    d) 4 times
    e) 5 times
    f) 6 times
    g) Not applicable

24. Numbness of a limb:
    a) 1 time
    b) 2 times
    c) 3 times
    d) 4 times
    e) 5 times
    f) 6 times
    g) Not applicable

25. Sensation of irregular heartbeat:
    a) 1 time
    b) 2 times
    c) 3 times
    d) 4 times
    e) 5 times
    f) 6 times
    g) Not applicable

26. Other non-specific chest symptoms during menstruation. Please provide a number from 1 to 6, or leave this field blank, if not applicable.

REGULARITY OF SYMPTOMS DURING OVULATION.

During the last 6 menstrual cycles, how many times did you experience the following symptoms during ovulation? Ovulation most commonly occurs on the 14th day of the menstrual cycle.

27. Chest pain:
    a) 1 time
    b) 2 times
    c) 3 times
    d) 4 times
    e) 5 times
    f) 6 times
    g) Not applicable

28. Dyspnoea (shortness of breath):
    a) 1 time
    b) 2 times
    c) 3 times
    d) 4 times

e) 5 times
f) 6 times
g) Not applicable

29. Cough:
    a) 1 time
    b) 2 times
    c) 3 times
    d) 4 times
    e) 5 times
    f) 6 times
    g) Not applicable

30. Haemoptysis (coughing up blood):
    a) 1 time
    b) 2 times
    c) 3 times
    d) 4 times
    e) 5 times
    f) 6 times
    g) Not applicable

31. Numbness of a limb:
    a) 1 time
    b) 2 times
    c) 3 times
    d) 4 times
    e) 5 times
    f) 6 times
    g) Not applicable

32. Sensation of irregular heartbeat:
    a) 1 time
    b) 2 times
    c) 3 times
    d) 4 times
    e) 5 times
    f) 6 times
    g) Not applicable

33. Other non-specific chest symptoms during ovulation. Please provide a number from 1 to 6, or leave this field blank, if not applicable.

ADDITIONAL QUESTION ABOUT CHEST PAIN DURING THE MENSTRUAL CYCLE.

If you have never experienced chest pain during the menstrual cycle, please skip this section.

34. Have you been diagnosed with cholelithiasis (gallstones)?
    a) Yes
    b) No

35. Does the chest pain typically occur after a meal?
    a) Yes
    b) No

36. Does the chest pain typically occur after a diet mistake?
    a) Yes
    b) No

THE NATURE OF CHEST PAIN DURING THE MENSTRUAL CYCLE.

In the following questions, please characterize your pain as accurately as possible by selecting one of the answers by choosing numbers from 0 to 4.

37. Pain intensity
    a) 0 – Without pain
    b) 1 – Mild
    c) 2 – Strong
    d) 3 – Very strong
    e) 4 – Unbearable

38. Pain frequency
    a) 0 – Pain does not occur
    b) 1 – Periodical
    c) 2 – Frequent
    d) 3 – Very frequent
    e) 4 – Continuous

39. Intake of painkillers
    a) 0 – No medications

b) 1 – Periodical
c) 2 – Permanent, small doses
d) 3 – Permanent, big doses
e) 4 – Permanent, very big doses

40. Limitation of physical activity
    a) 0 – None
    b) 1 – Partial
    c) 2 – Demands partial help/makes work difficult
    d) 3 – Demands partial help/makes work impossible
    e) 4 – Demands full-time help/prevents independent functioning

41. What aggravates the pain?

42. What alleviates the pain?

PAIN LOCATION.

In this section, we kindly ask you to mark on the diagram all the areas that are painful during the menstrual cycle. Please do not be influenced by the borders already marked on the diagram.

If possible, please use a mobile device (computer, phone, tablet) as follows:

1. Click on the link below and download the file.
2. Mark the relevant areas on the diagram and save the file.
3. Upload your file below ("Add file").
4. Exit the file uploading window ("✓ Complete").

For those who need assistance in editing the diagram:
It can be done in several simple ways by:
- editing the PDF file directly
- saving the file as a Microsoft Word document - then use the 'Draw' function
- taking a screenshot of the diagram - such a screenshot can be edited via various applications (Microsoft Paint, Messenger, etc.)

QUESTIONNAIRE – POLISH VERSION

Jesteśmy grupą badawczą z Gdańskiego Uniwersytetu Medycznego prowadzącą badanie na temat bólu w endometriozie klatki piersiowej. Za pomocą stworzonej przez nas ankiety chcemy zebrać dane kliniczne oraz w oparciu o nie opracować publikację, opisującą to zjawisko.
Przyświecającym nam celem jest usprawnienie procesu diagnostycznego endometriozy klatki piersiowej oraz zwiększanie świadomości na temat tej choroby, zarówno wśród pacjentek, jak i klinicystów.

W związku z powyższym, zwracamy się do Państwa z uprzejmą prośbą o wypełnienie ankiety - badanie jest w pełni anonimowe, a całość powinna zająć max. 10-15 minut. Głęboko wierzymy, iż razem możemy przyczynić się do poprawy jakości życia wielu pacjentek na całym świecie.

Przed rozpoczęciem ankiety prosimy o zapoznanie się z poniższymi definicjami endometriozy:
1. Endometrioza jest określana jako obecność ektopowej tkanki endometrium (gruczołów oraz zrębu) poza jamą macicy.
2. Endometrioza klatki piersiowej obejmuje zmiany w płucach, jamie opłucnej, przeponie i oskrzelach potwierdzone za pomocą badań histopatologicznych z wycinków (pobranych na drodze aspiracji, torakotomii lub bronchoskopii).

W wypadku problemów technicznych lub pytań w sprawie ankiety zapraszamy do kontaktu na adres mailowy:endometriozaankieta@gmail.com

1. Ile ma Pani lat? Proszę podać samą liczbę.

2. Czy odczuwa Pani bóle w klatce piersiowej podczas cyklu miesiączkowego?
   a) Tak
   b) Nie

3. Po której stronie odczuwa Pani bóle w klatce piersiowej podczas cyklu miesiączkowego?
   a) Po prawej stronie
   b) Po lewej stronie
   c) Po obu stronach
   d) Nie dotyczy

4. Czy została u Pani zdiagnozowana endometrioza?
   a) Tak
   b) Nie

5. Czy została u Pani zdiagnozowana endometrioza klatki piersiowej?
   a) Tak
   b) Nie

6. Czy została u Pani zdiagnozowana endometrioza przepony?
   a) Tak
   b) Nie

7. W jaki sposób została postawiona diagnoza endometriozy?
   a) Śródoperacyjnie
   b) Radiologicznie
   c) Inny test
   d) Nie dotyczy

8. Ile miała Pani lat kiedy została postawiona diagnoza? Proszę podać samą liczbę. Jeśli nie ma Pani diagnozy endometriozy, proszę zostawić to pole puste.

9. Czy w Pani rodzinie zdarzyły się potwierdzone przypadki endometriozy?
   a) Tak
   b) Nie

PYTANIA DOTYCZĄCE PŁODNOŚCI.

10. Czy kiedykolwiek miała Pani problemy z zajściem w ciążę?
    a) Tak
    b) Nie
    c) Nie podejmowałam starań

11. Czy była Pani leczona z powodu niepłodności?
    a) Tak
    b) Nie

12. Ile razy była Pani w ciąży?

13. Ile razy doświadczyła Pani poronienia?

TERAPIA HORMONALNA.

14. Czy stosuje Pani terapię hormonalną?
    a) Tak
    b) Nie

15. Jeśli tak – od jakiego czasu?

16. Czy przeszła Pani któryś z poniższych zabiegów?
    a) Histerektomia (usunięcie macicy)
    b) Adneksetomia (usunięcie jajowodów i jajników)
    c) Żaden z powyższych

OBJAWY WYSTĘPUJĄCE PODCZAS CYKLU MIESIĘCZNEGO.

Jeśli stosuje Pani leczenie hormonalne oraz:

a) nie ma objawów ⇨ prosimy odnieść się do okresu pełnoobjawowego, sprzed leczenia hormonalnego

b) nadal ma obawy ⇨ prosimy odnieść się do objawów aktualnych

17. Czy w ciągu ostatnich 6 cykli miesiączkowych doświadczyła Pani któregokolwiek z poniższych objawów? Proszę zaznaczyć wszystkie, które Pani dotyczą.
    a) Ból w klatce piersiowej
    b) Duszność
    c) Kaszel
    d) Krwioplucie
    e) Uczucie ogłuszonej kończyny
    f) Uczucie niemiarowej akcji serca
    g) Nie dotyczy

18. Inne niecharakterystyczne objawy w klatce piersiowej w ciągu ostatnich 6 cykli miesiączkowych (np. uczucie "pykania", przelewania się płynu). Jeśli to Pani nie dotyczy, proszę zostawić to pole puste.

19. W jakim wieku zaczęły się u Pani powyższe objawy? Proszę podać samą liczbę. Jeśli te objawy Pani nie dotyczą, proszę zostawić to pole puste.

REGULARNOŚĆ OBJAWÓW PODCZAS MENSTRUACJI.

W ciągu ostatnich 6 cykli miesiączkowych, podczas ilu miesiączek (okresów krwawienia) doświadczyła Pani poniższych objawów? Następna sekcja dotyczyć będzie owulacji, dlatego prosimy, aby w tej części skupić się wyłącznie na okresach krwawienia.

20. Ból w klatce piersiowej:
    a) 1 raz
    b) 2 razy
    c) 3 razy
    d) 4 razy
    e) 5 razy
    f) 6 razy
    g) Nie dotyczy

21. Duszność:
    a) 1 raz
    b) 2 razy
    c) 3 razy
    d) 4 razy
    e) 5 razy
    f) 6 razy
    g) Nie dotyczy

22. Kaszel:
    a) 1 raz
    b) 2 razy
    c) 3 razy
    d) 4 razy
    e) 5 razy
    f) 6 razy
    g) Nie dotyczy

23. Krwioplucie:
    a) 1 raz
    b) 2 razy
    c) 3 razy
    d) 4 razy
    e) 5 razy
    f) 6 razy
    g) Nie dotyczy

24. Uczucie ogłuszonej kończyny:
    a) 1 raz
    b) 2 razy
    c) 3 razy
    d) 4 razy
    e) 5 razy
    f) 6 razy
    g) Nie dotyczy

25. Uczucie niemiarowej akcji serca:
    a) 1 raz
    b) 2 razy
    c) 3 razy
    d) 4 razy
    e) 5 razy
    f) 6 razy
    g) Nie dotyczy

26. Inne niecharakterystyczne objawy w klatce piersiowej podczas miesiączki. Proszę podać liczbę od 1 do 6 lub zostawić to pole puste, jeśli wcześniej nie podała Pani żadnych objawów.

REGULARNOŚĆ OBJAWÓW PODCZAS OWULACJI.

W ciągu ostatnich 6 cykli miesięcznych, podczas ilu owulacji doświadczyła Pani poniższych objawów? Do owulacji dochodzi najczęściej 14 dnia cyklu.

27. Ból w klatce piersiowej:
    a) 1 raz
    b) 2 razy
    c) 3 razy
    d) 4 razy
    e) 5 razy
    f) 6 razy
    g) Nie dotyczy

28. Duszność:
    a) 1 raz
    b) 2 razy
    c) 3 razy

d) 4 razy
e) 5 razy
f) 6 razy
g) Nie dotyczy

29. Kaszel:
    a) 1 raz
    b) 2 razy
    c) 3 razy
    d) 4 razy
    e) 5 razy
    f) 6 razy
    g) Nie dotyczy

30. Krwioplucie:
    a) 1 raz
    b) 2 razy
    c) 3 razy
    d) 4 razy
    e) 5 razy
    f) 6 razy
    g) Nie dotyczy

31. Uczucie ogłuszonej kończyny:
    a) 1 raz
    b) 2 razy
    c) 3 razy
    d) 4 razy
    e) 5 razy
    f) 6 razy
    g) Nie dotyczy

32. Uczucie niemiarowej akcji serca:
    a) 1 raz
    b) 2 razy
    c) 3 razy
    d) 4 razy
    e) 5 razy
    f) 6 razy
    g) Nie dotyczy

33. Inne niecharakterystyczne objawy w klatce piersiowej podczas owulacji. Proszę podać liczbę od 1 do 6 lub zostawić to pole puste, jeśli wcześniej nie podała Pani żadnych objawów.

PYTANIA UZUPEŁNIAJĄCE O BÓL W KLATCE PIERSIOWEJ PODCZAS CYKLU MIESIĘCZNEGO.

Jeżeli nigdy nie doświadczyła Pani bólu w klatce piersiowej podczas cyklu miesięcznego, proszę pominąć tą sekcję.

34. Czy została u Pani zdiagnozowana kamica pęcherzyka żółciowego?
    a) Tak
    b) Nie

35. Czy ból w klatce piersiowej pojawia się zazwyczaj po posiłku?
    a) Tak
    b) Nie

36. Czy ból w klatce piersiowej pojawia się zazwyczaj po błędzie dietetycznym?
    a) Tak
    b) Nie

CHARAKTER BÓLU W KLATCE PIERSIOWEJ PODCZAS CYKLU MIESIĘCZNEGO.

W poniższych pytaniach proszę scharakteryzować towarzyszący ból wybierając punkty od 0 do 4.

37. Nasilenie Bólu
    a) 0 - Bez bólu
    b) 1 – Łagodny
    c) 2 – Silny
    d) 3 – Bardzo silny
    e) 4 – Nie do wytrzymania

38. Częstotliwość występowania bólu
    a) 0 – Nie występuje
    b) 1 – Okresowo
    c) 2 – Często
    d) 3 – Bardzo często
    e) 4 – Ból ciągły

39. Częstotliwość zażywania środków przeciwbólowych
    a) 0 – Brak środków przeciwbólowych
    b) 1 – Doraźnie
    c) 2 – Ciągle, małe dawki
    d) 3 – Ciągle, duże dawki
    e) 4 – Ciągle, bardzo duże dawki

40. Ograniczenie aktywności ruchowej
    a) 0 – Żadne
    b) 1 – Częściowe
    c) 2 – Utrudniające pracę
    d) 3 – Uniemożliwiające pracę
    e) 4 – Uniemożliwiające samodzielne funkcjonowanie

41. Co nasila ból?

42. Co łagodzi ból?

LOKALIZACJA BÓLU.
W tym miejscu prosimy Panią o zaznaczenie na schemacie wszystkich obszarów, w których odczuwa Pani ból podczas cyklu miesięcznego. Proszę nie sugerować się granicami obszarów już zaznaczonych na schemacie.

Jeżeli jest to możliwe, prosimy zrobić to na urządzeniu mobilnym (komputer, telefon, tablet) w następującym sposób:

1. Kliknąć w poniższy link i pobrać plik.
2. Zaznaczyć odpowiednie obszary na schemacie i zapisać plik.
3. Dodać swój plik w odpowiednim miejscu poniżej ("Add file”).
4. Opuścić okno przesyłania pliku, klikając przycisk "Ukończono".

Dla osób, które potrzebują pomocy w edycji schematu:
Można tego dokonać na kilka prostych sposobów:
- bezpośrednio edytując plik PDF
- zapisując plik jako dokument Microsoft Word - tam można skorzystać z funkcji „Rysowanie”
- robiąc zrzut ekranu schematu - taki zrzut można edytować za pomocą różnych aplikacji, również na telefonie (Microsoft Paint, Messenger etc.)
